# Biomechanical Analysis of Stand-alone Lateral Lumbar Interbody Fusion for Lumbar Adjacent Segment Disease

**DOI:** 10.7759/cureus.6208

**Published:** 2019-11-20

**Authors:** Michael Chioffe, Michael McCarthy, Peter R Swiatek, Joseph P Maslak, Leonard I Voronov, Robert M Havey, Muturi Muriuki, Avinash Patwardhan, Alpesh A Patel

**Affiliations:** 1 Orthopaedic Surgery, Sarah Bush Lincoln Health Center, Mattoon, USA; 2 Orthopaedics, Spine Surgery, Hospital for Special Surgery, New York, USA; 3 Orthopaedic Surgery, Northwestern Memorial Hospital, Chicago, USA; 4 Orthopaedics, Spine Surgery, Cleveland Clinic, Cleveland, USA; 5 Orthopaedic Surgery, Loyola University Chicago, Maywood, USA; 6 Orthopaedic Surgery, Edward Hines, Jr. Veterans Administration Hospital, Hines, USA; 7 Orthopaedic Surgery, Loyola University Medical Center, Chicago, USA

**Keywords:** interbody fusion, posterior spinal fusion, adjacent segment disease, spondylosis, biomechanics

## Abstract

Study design

Biomechanical cadaveric study

Objective

To compare biomechanical properties of a single stand-alone interbody fusion and a single-level pedicle screw construct above a previous lumbar pedicle fusion.

Summary of background data

Adjacent segment disease (ASD) is spondylosis of adjacent vertebral segments after previous spinal fusion. Despite the consensus that ASD is clinically significant, the surgical treatment of ASD is controversial.

Methods

Lateral lumbar interbody fusion (LLIF) and posterior spinal fusion (PSF) with pedicle screws were analyzed within a validated cadaveric lumbar fusion model. L3-4 vertebral segment motion was analyzed within the following simulations: without implants (intact), L3-4 LLIF-only, L3-4 LLIF with previous L4-S1 PSF, L3-4 PSF with previous L4-S1 PSF, and L4-S1 PSF alone. L3-4 motion values were measured during flexion/extension with and without axial load, side bending, and axial rotation.

Results

L3-4 motion in the intact model was found to be 4.7 ± 1.2 degrees. L3-4 LLIF-only decreased motion to 1.9 ± 1.1 degrees. L3-4 LLIF with previous L4-S1 fusion demonstrated less motion in all planes with and without loading (p < 0.05) compared to an intact spine. However, L3-4 motion with flexion/extension and lateral bending was noted to be greater compared to the L3-S1 construct (p < 0.5). The L3-S1 PSF construct decreased motion to less than 1° in all planes of motion with or without loading (p < 0.05). The L3-4 PSF with previous L4-S1 PSF constructs decreased the flexion/extension motion by 92.4% compared to the intact spine, whereas the L3-4 LLIF with previous L4-S1 PSF constructs decreased motion by 61.2%.

Conclusions

Stand-alone LLIF above a previous posterolateral fusion significantly decreases motion at the adjacent segment, demonstrating its utility in treating ASD without necessitating revision. The stand-alone LLIF is a biomechanically sound option in the treatment of ASD and is advantageous in patient populations who may benefit from less invasive surgical options.

## Introduction

Adjacent segment disease (ASD) is a postoperative diagnosis in patients with prior histories of spinal fusion. A diagnosis of ASD is predicated on both adjacent segment radiographic degeneration and the development of clinical symptoms. Multiple longitudinal studies have demonstrated that radiographic evidence of adjacent segment degeneration is not directly correlated with ASD, as some radiographic findings can be nonspecific and not clinically relevant [1-3]. Numerous studies have identified multiple risk factors for the development of ASD, including age over 60 years, male gender, facet degeneration, multilevel fusion, fusion to L5, and preexisting degenerative disc disease adjacent to the fused segment [4-8].

Treatment of symptomatic ASD traditionally includes surgical decompression and extension of the existing posterior instrumentation and fusion. The approach to revising posterior spinal fusions is often more complex than the index procedure and has been associated with increased complications and poor outcomes [2, 9-10]. The difficulty of revision posterior spinal fusion often leads to the use of minimally invasive interbody fusion. Anterior, oblique, and lateral retroperitoneal approaches have become increasingly popular for the treatment of symptomatic ASD [11-12]. The direct lateral approach is an effective means for both successful arthrodesis and indirect decompression [11, 13-14].

While the use of lateral lumbar interbody fusion (LLIF) for ASD has been reported in recent literature, LLIF alone has not been rigorously investigated in a cadaveric model [15-16]. The adjacent segment is a difficult biomechanical environment for fusion with increased stresses and abnormal motion patterns due to the lack of mobility. The purpose of this study was to compare biomechanical properties of a single stand-alone interbody fusion and a single-level pedicle screw construct above a previous lumbar pedicle fusion.

## Materials and methods

Six fresh-frozen human lumbar spine specimens (T10-S1) were used for this study with the demographics of each listed in Table 1.

**Table 1:** Specimen Demographics GSW: gunshot wound; SD: standard deviation

Specimen	Age (years)	Sex	Cause of Death
1	52	M	Respiratory Distress Syndrome
2	39	M	Lung Cancer
3	44	M	Cirrhosis of the Liver
4	49	F	Drug Overdose
5	51	F	Diabetic Shock
6	32	M	GSW to the Head
Mean	44.5		
SD	7.8		

Radiographic screening was performed to exclude specimens with fractures, metastatic disease, bridging osteophytes, osteoporosis, previous spine surgeries, or other conditions that could significantly affect the biomechanics of the spine. The specimen was thawed and stripped of the paraspinal musculature while preserving the discs, facet joints, and osteoligamentous structures.

**Figure 1:**
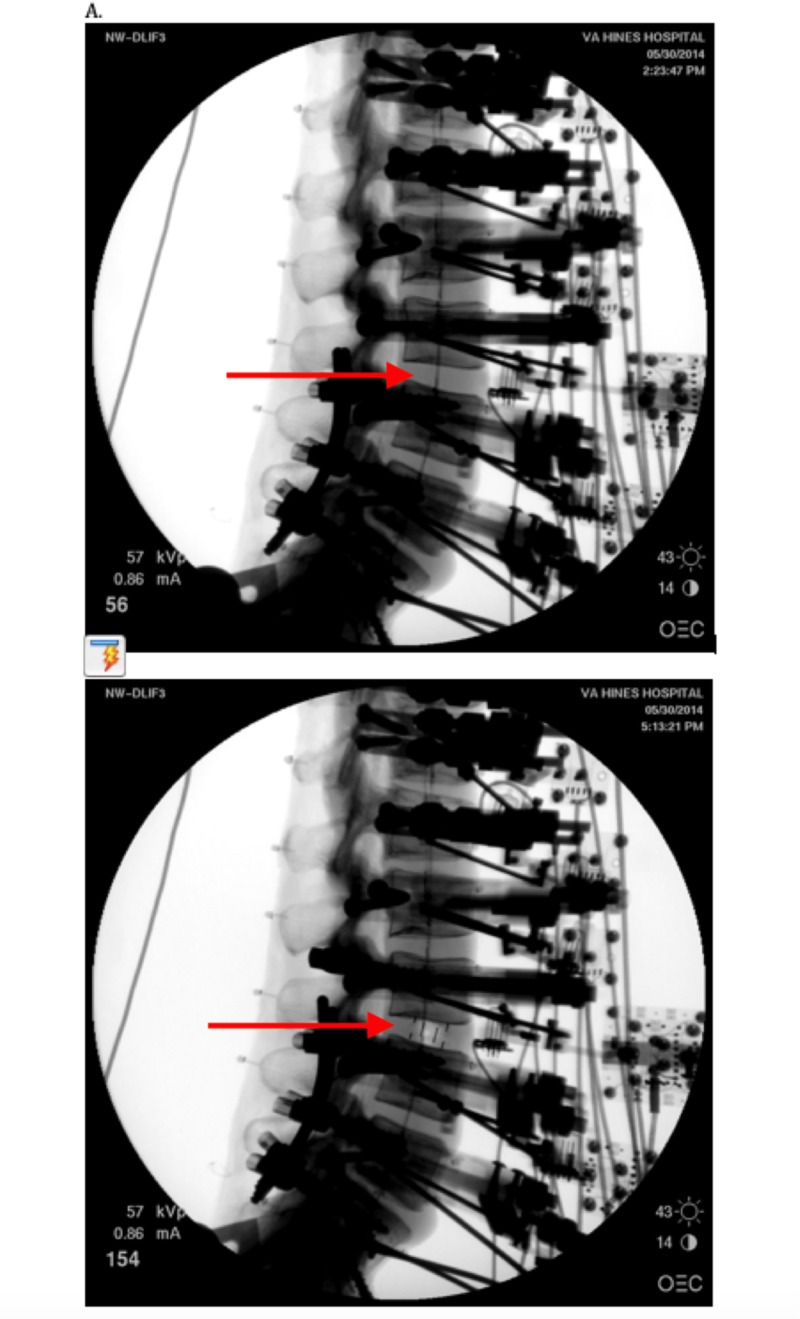
Radiographic images before and after interbody spacer placement A) X-ray images of the cadaver spine specimen before interbody spacer placement; B) X-ray images of the cadaver spine specimen after interbody spacer placement

All biomechanical testing was performed at room temperature. The specimen was fixed to the apparatus at the caudal end and free to move in any plane at the proximal end. The apparatus allowed continuous cycling of the specimen between specified maximum moment endpoints in flexion-extension, lateral bending, and axial rotation (Figure 1).

The load-displacement data was collected until two reproducible load-displacement loops were obtained.

**Figure 2:**
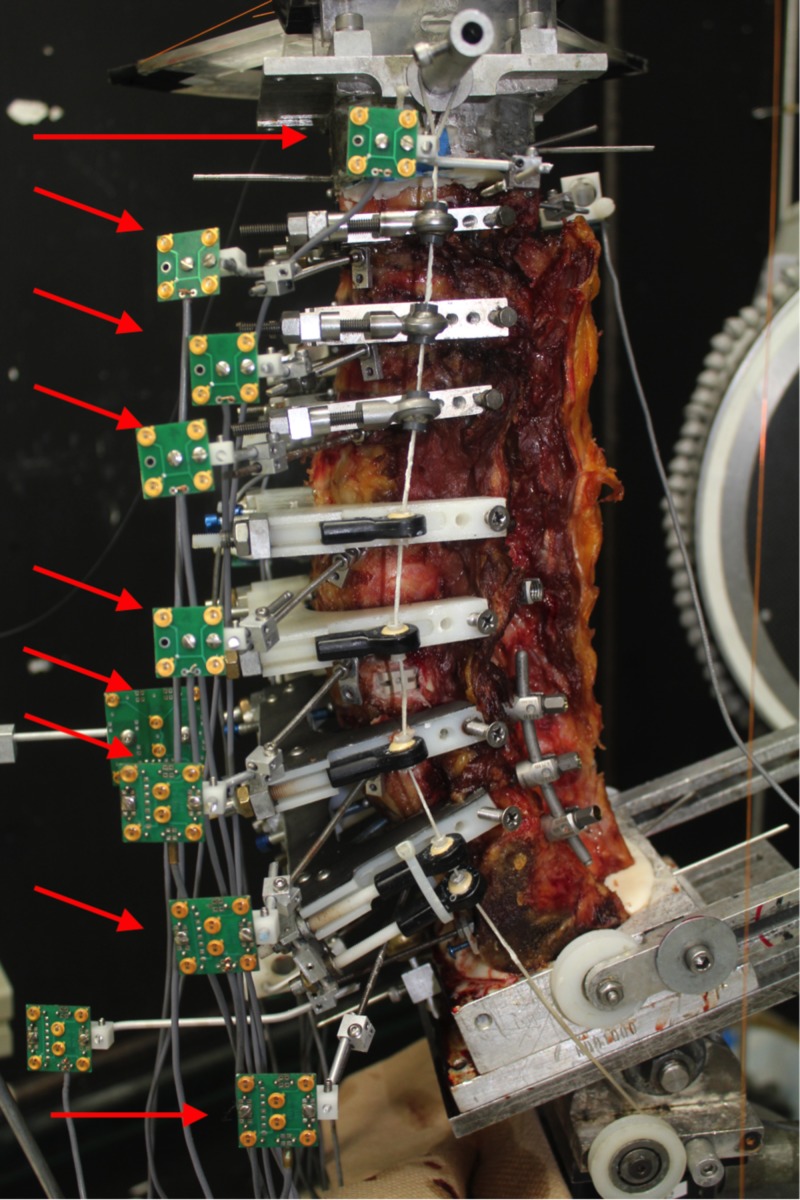
Photograph of the lumbar spine specimen with motion analysis sensors in place

The angular motion of the T10 to S1 vertebrae was measured using an optoelectronic motion measurement system (Optotrak Certus®, Northern Digital, Inc., Waterloo, Ontario, Canada) (Figure 2). 

In addition, biaxial angle sensors (Model 902-45 Biaxial Clinometer) (Applied Geomechanics, Inc., Santa Cruz, CA) were mounted on each vertebra to allow real-time feedback for the optimization of the preload path. A six-component load cell (Model MC3A-6-1000) (AMTI, Inc., Watertown, MA) was placed under the specimen to measure the applied compressive preload and moments. Fluoroscopic imaging (General Electric OEC® 9800 Plus digital fluoroscopy machine) (GE Healthcare, Chicago, IL) was used during implantation to ensure proper implant placement. Fluoroscopic images of the mobile segments were taken in the neutral, flexed, and extended postures during the kinematic testing.

Each specimen’s intact range of motion (ROM) was tested in flexion-extension, lateral bending, and axial rotation under moment control using 8 N (newtons) flexion and 6 N extension moments with no preload and then with 400 N following preload. Each specimen was tested using the following stepwise protocol: intact, L4-S1 posterior spinal fusion (PSF), L3-S1 PSF, L4-S1 PSF with LLIF at L3-4, then LLIF at L3-4 without PSF. Following intact testing, a fusion was performed at L4-S1 using posterior pedicle screws with rods. Bilateral pedicle screws and rods were then extended to the L3-L4 segment and the previous load control protocol was repeated. Next, the lateral interbody cage was implanted at the L3-L4 segment and the previous load control protocol was repeated. The lateral technique involved the preservation of the anterior longitudinal ligament but with the release of the ipsilateral and contralateral annulus. The final step of the protocol was testing a stand-alone LLIF at L3-L4 after removing posterolateral fusion.

## Results

Following posterolateral fusion at L4-S1, the ROM at L3-L4 increased compared to the intact condition in flexion-extension, lateral bending, and axial rotation but did not reach statistical significance (p > 0.05) (Table 2). 

**Table 2:** The L3-L4 Range of Motion Values (Mean ± SD) During Flexion-Extension (0N and 400N), Lateral Bending, and Axial Rotation (* denotes significant difference from intact, p < 0.05) († denotes significant difference from L3-S1 fusion, p < 0.05) DLIF: direct lateral interbody fusion; Ext: extension; Flex: flexion; N: newtons; ROM: range of motion; SD: standard deviation

L3-L4 ROM (degrees)	Intact	L4-S1 Fusion	L3-S1 Fusion	L4-S1 Fusion, DLIF at L3-L4	Stand-alone DLIF at L3-L4
Flex-Ext 0N	4.7 ± 1.2	5.4 ± 1.3	0.5 ± 0.2*	2.0 ± 0.9*†	1.9 ± 1.1
Flex-Ext 400N	5.2 ± 1.0	5.7 ± 1.1	0.4 ± 0.1*	2.1 ± 0.8*†	1.8 ± 1.0
Lateral Bending	8.2 ± 1.7	8.3 ± 1.7	0.6 ± 0.1*	4.0 ± 1.8*†	4.6 ± 2.0
Axial Rotation	1.4 ± 0.7	1.3 ± 0.8	0.5 ± 0.2*	0.7 ± 0.3*	1.3 ± 0.8

When the posterolateral fusion was extended to L3, there was a significant decrease in L3-L4 motion (less than one degree) in all-loading modes (p < 0.05). When posterolateral fusion at L3-L4 was replaced by a stand-alone LLIF (above a fusion at L4-S1), the ROM at L3-L4 continued to be significantly lower than intact (p < 0.05 in all loading modes) but was also significantly greater than that of a posterolateral fusion at L3-L4 (p < 0.05 for flexion-extension and lateral bending, p = 0.11 for axial rotation). Overall, the L3-S1 pedicle screw fusion constructs decreased the flexion-extension ROM at 400 N by 92.0%, whereas the L3-4 LLIF and L4-S1 fusion construct decreased flexion-extension ROM by 58.6% of intact. Removal of a posterolateral fusion at L4-S1, leaving a stand-alone direct lateral interbody fusion (DLIF) at L3-L4, did not significantly change the motion at L3-L4 (p > 0.05).

## Discussion

The adjacent level of a prior posterior spinal fusion is a biomechanically and biologically challenging environment [17]. The lateral approach offers a minimally invasive method of fusion while avoiding the complications associated with revision posterior spinal fusion [18]. In addition to avoiding these complications, the LLIF can address underlying foraminal and central stenosis via indirect decompression [8, 19]. Recent studies have demonstrated the capabilities of lateral interbody fusion in the treatment of ASD [6, 20-21]. LLIF is an attractive alternative in treating ASD due to limited blood loss, no need to reexplore previous laminectomies, and an overall reduction in complications [6]. Although LLIF has been demonstrated to be a viable alternative to PSF, there still lacks a significant body of literature supporting its use as the gold standard in treating ASD [22]. 

This biomechanical study demonstrates a statistically significant reduction in motion in all load parameters and all axes of motion with a stand-alone lateral interbody fusion adjacent to a prior posterolateral fusion. Although our model demonstrated less rigidity (56% loss of motion) with the interbody construct compared to the posterior instrumented fusion (92% loss of motion), it remains an attractive option in the treatment of ASD.

Within our study, the results of extending the PSF in the treatment of ASD provide evidence of this construct’s ability to provide increased structural rigidity. Our outcomes demonstrate a significant decrease in L3-L4 motion in all-loading modes (p < 0.05); however, this study did not assess the significance of this increased stability in comparison to the LLIF construct. Results demonstrated that the LLIF construct provides a significant reduction in motion compared to an intact spine; however, this study was unable to assess superiority between revision PSF and LLIF. The tenants of orthopedic surgery and spine fusion surgery are predicated on reducing motion, thus increasing a construct’s ability to aid in fusion; therefore, increased rigidity can be correlated with a likely increased fusion mass. Assessing the association between rigidity and likelihood of fusion is outside the scope of this study; thus, one limitation of our study is its inability to quantify significant motion reduction to a fusion between the two constructs.

This study confirms the stability provided by a stand-alone LLIF construct for the treatment of adjacent segment disease above a previous lumbar fusion. In addition to avoiding the complication associated with posterior revision surgery, the LLIF’s rigidity and large intervertebral fusion bed make it an attractive option in the treatment of ASD. Recent studies have demonstrated LLIF’s viability as a treatment modality for ASD; however, large scale prospective studies are needed to further delineate potential benefits and indications for use of this stand-alone interbody construct for this disease [15, 20].

## Conclusions

Recent advancements with the implementation of interbody fusion constructs and their versatility within spinal surgery have made for an attractive option in minimally invasive spine fusion. In the setting of ASD, interbody constructs, specifically, LLIF, are powerful tools for surgeons in need of fusion options while avoiding the complications associated with posterior revision surgery. Our study provides in vitro biomechanical evidence demonstrating a significant reduction of motion at the level adjacent to a prior posterior spinal fusion. The LLIF is a biomechanically sound option for fusion in the treatment of ASD and is advantageous in patient populations who would benefit from minimally invasive surgery.
